# Collapsing Focal Segmental Glomerulosclerosis With Anti-nephrin Antibody Following Minimal Change Disease Possibly Triggered by COVID-19 Infection: A Case Report

**DOI:** 10.7759/cureus.96622

**Published:** 2025-11-11

**Authors:** Hideyo Oguchi, Yoko Shirai, Kenichiro Miura, Motoshi Hattori, Yuko Hamasaki, Masaki Muramatsu, Kazuho Honda, Tetuo Mikami, Naobumi Tochigi, Ken Sakai

**Affiliations:** 1 Deparment of Nephrology, Faculty of Medicine, Toho University, Tokyo, JPN; 2 Department of Pediatric Nephrology, Tokyo Women's Medical University, Tokyo, JPN; 3 Department of Nephrology, Faculty of Medicine, Toho University, Tokyo, JPN; 4 School of Medicine, Showa University, Tokyo, JPN; 5 Department of Pathology, Faculty of Medicine, Toho University, Tokyo, JPN

**Keywords:** anti-nephrin antibody, collapsing fsgs, covid-19 infection, fsgs, minimal change disease

## Abstract

A man in his forties experienced foamy urine after COVID-19 infection and received a kidney biopsy. He received tacrolimus for myasthenia gravis; no kidney disease was noted before COVID-19 infection, and he had no family history of kidney disease. The biopsy showed minimal change disease, characterized by IgG deposition in the podocyte and mesangial areas. Prednisone was started at 70 mg, but he required hemodialysis three months after COVID-19 infection. A second biopsy 15 months after the COVID-19 infection showed collapsing focal segmental glomerulosclerosis (FSGS) with IgG deposition in the podocytes and mesangium. Immunofluorescence imaging of the second biopsy using structure illumination microscopy showed decreased nephrin expression and punctuate IgG deposition colocalized with nephrin. Thereafter, his serum was positive for anti-nephrin antibodies (367 IU/ml, cut-off 226 U/ml). To the best of our knowledge, no other reports of collapsing FSGS or minimal change disease with anti-nephrin antibodies triggered by COVID-19 infection have been published. This case summarizes a novel case of collapsing FSGS with IgG-positive podocytes associated with anti-nephrin antibodies following minimal change disease, possibly triggered by COVID-19 infection. Further study is needed to clarify the mechanism by which anti-nephrin antibodies are produced by COVID-19 infection.

## Introduction

Minimal change disease and idiopathic focal segmental glomerulosclerosis (FSGS) are the main pathological findings of idiopathic nephrotic syndrome and have been reported to have a common etiology [1]. Circulating permeability factors may be involved in the pathogenesis of minimal change disease and FSGS [2]. For example, Watts et al. discovered that circulating anti-nephrin antibodies were related to the etiology of minimal change disease and that punctuate IgG in podocytes correlated with anti-nephrin antibodies [3]. In a recent multicenter observational study, Shirai et al. reported a possible role for circulating anti-nephrin antibodies in FSGS recurrence after kidney transplantation [4].

A recent systematic review of biopsies taken from patients with COVID-19 showed various acute and chronic histological findings of the glomerulus, and vascular and tubule interstitial change [5]. They found that collapsing FSGS was the commonest glomerular lesion with a prevalence of 54%, whereas non-collapsing FSGS and minimal change disease had a prevalence of 2% each [5]. To the best of our knowledge, there are currently no reports on the association between anti-nephrin antibodies and collapsing FSGS or minimal change disease after COVID-19 infection. Here, we present a unique case of collapsing FSGS with IgG-positive staining in podocytes related to anti-nephrin antibodies following minimal change disease, possibly triggered by COVID-19 infection.

## Case presentation

A Japanese man in his forties had a COVID-19 infection and experienced foamy urine one month later. He was taking tacrolimus for myasthenia gravis; no kidney disease was noted before COVID-19 infection, and he had no family history of kidney disease. He had nephrotic syndrome and received a renal biopsy at a hospital in the United States. A biopsy showed minimal change disease, and IgG deposition was found in the podocyte and mesangium areas. Electron microscopy showed diffuse foot process effacement. Prednisone was initiated at 70 mg. However, he required hemodialysis three months after the COVID-19 infection, and he received rituximab therapy eight months after the infection. A second renal biopsy was performed at our hospital 15 months after the infection. Laboratory results at the time of biopsy are shown in Table 1. The patient still had nephrotic syndrome with low serum albumin (1.7 g/dl) and proteinuria (24.9 g/gCr).

**Table 1:** Laboratory data at the time of the second biopsy WBC: white blood cells; RBC: red blood cell; Hb: hemoglobin; Cr: creatinine; eGFR: estimated glomerular filtration rate; TC: total cholesterol; LDL-C: low density lipoprotein cholesterol; HDL-C: high density lipoprotein cholesterol; GOT: glutamic oxaloacetic transaminase; GPT: glutamic pyruvic transaminase; Plt: platelet; TP: total protein

Parameter	Value	Reference range	Parameter	Value	Reference range
WBC, /μl	8800	3300-8600	IgG, mg/dl	175	861-1747
RBC, /μl	389 × 10^4 ^	435 × 10^4^-555 × 10^4^	IgA, mg/dl	140	93-393
Hb, g/dl	12.1	13.7-16.8	IgM, mg/dl	143	33-183
Plt, /μl	204 × 10^3^	158 × 10^3^-348 × 10^3^	IgE IU/ml	102	≤173
TP, g/dl	4.0	6.6-8.1	ANA	negative	
Alb, g/dl	1.7	4.1-5.1	Anti-ds DNA IgG, IU/ml	<10	<12
BUN, mg/dl	28	8-20	MPO-ANCA, U/ml	<1.0	<3.5
Cr mg/dl	7.75	0.65-1.07	Anti-GBM antibody, U/ml	<1.5	≤7.0
eGFR ml/min/1.73m^2^	7.0		Urinary analysis		
GOT, U/l	12	13-30	Urine glucose	(2+)	
GPT, U/l	5	10-42	Urine protein	(3+)	
LDL-C, mg/dl	212	65-139	Urine blood	(3+)	
HDL-C, mg/dl	71	40-90	Urine RBC	30-49/1F	
Na, mmol/l	146	138-145	Urine WBC	10-19/1F	
K, mmol/l	3.6	3.6-4.8	Urine granular cast	(+)	
Cl, mmol/l	111	101-108	Urine fatty cast	(+)	
Ca, mg/dl	7.9	8.8-10.1	Urine protein	24.9 g/gCr	
IP, mg/dl	6.4	2.7-4.6			

**Figure 1:**
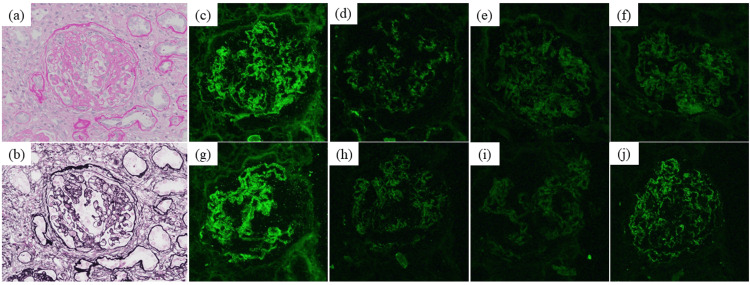
Collapsing FSGS features under a light microscope and immunofluorescence staining FSGS: focal segmental glomerulosclerosis; PAS: periodic acid-Schiff; PAM: periodic acid-methenamine silver (a) PAS and (b) PAM staining (original magnification ×400), (c)-(j) immunofluorescence staining, (c) IgG, (d) C3, (e) kappa, (f) lambda, (g) IgG1, (h) IgG2, (i) IgG3, and (j) IgG4

**Figure 2:**
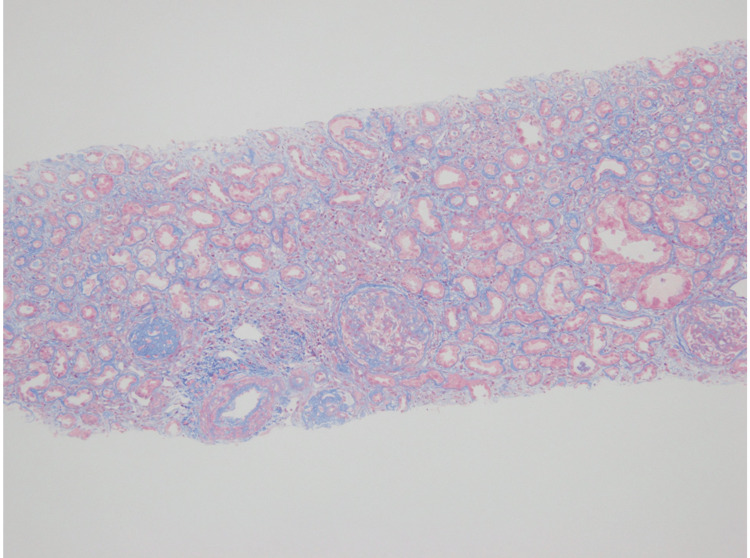
Light microscopic image (Masson’s trichrome stain, original magnification ×100)

**Figure 3:**
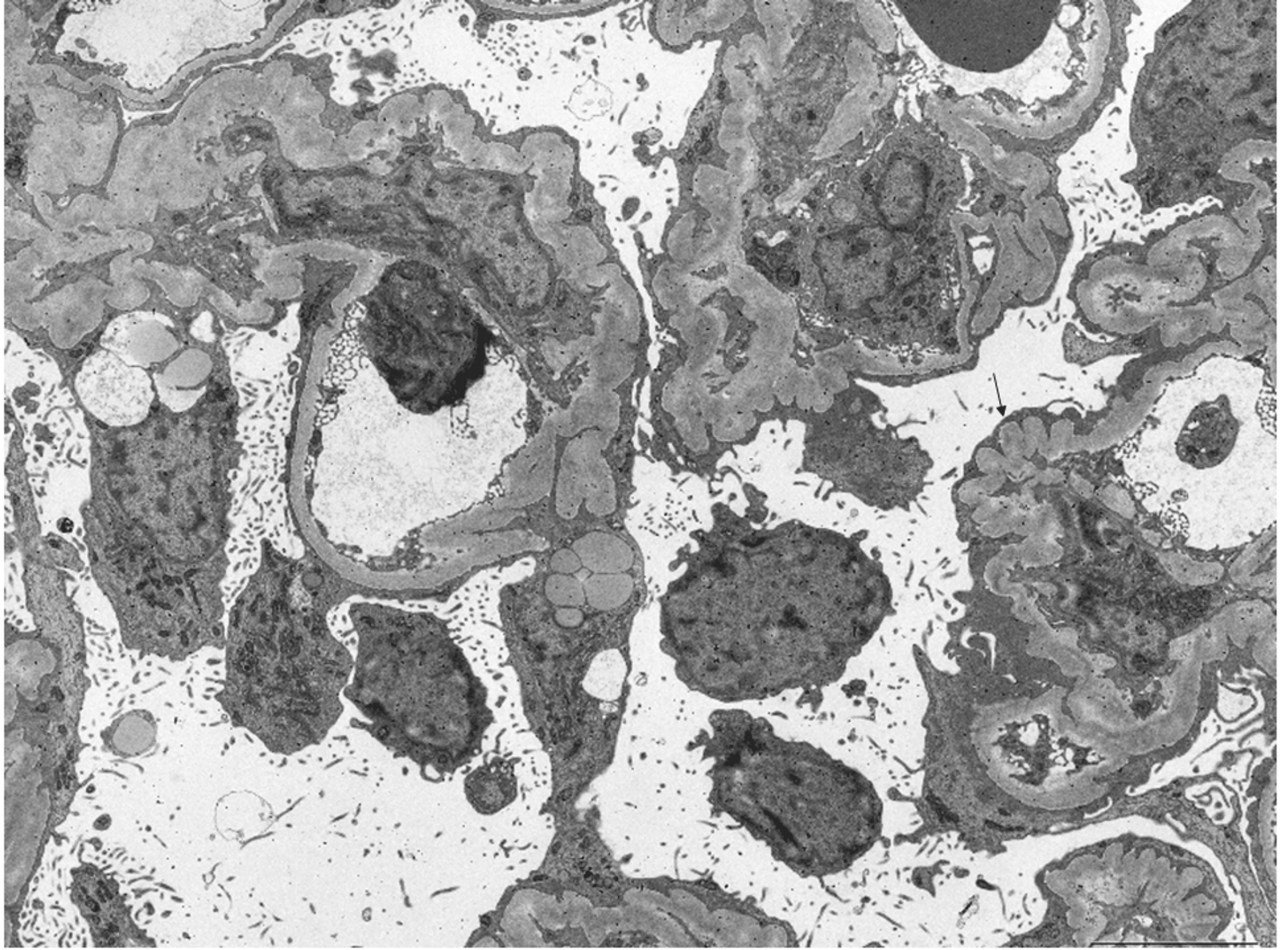
Electron microscopic image Diffuse podocyte foot process effacement was observed (black arrow)

**Figure 4:**
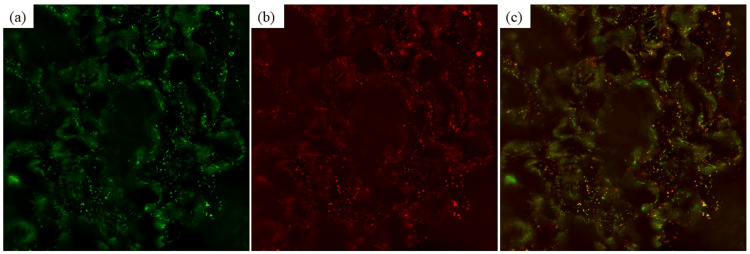
Structure illumination microscopy image (original magnification ×1000) (a) IgG, (b) nephrin, and (c) merged image (IgG + nephrin)

The pathological features of the biopsy are shown in Figure 1. Light microscopy of the biopsy indicated nine glomeruli, of which two showed global glomerulosclerosis and two showed segmental sclerosis. One glomeruli of segmental sclerosis showed glomerular capillary collapse, with epithelial cell hyperplasia, indicative of collapsing FSGS (Figures 1a, 1b) [6]. Tubular atrophy and interstitial fibrosis were severe (>50%) according to the Banff criteria of transplant pathology (Figure 2) [7]. Immunofluorescence staining showed the following intensities: IgG (+) in podocytes and mesangium (Figure 1c), IgA negative, IgM negative, C3 (±) in mesangium (Figure 1d) and C1q negative; kappa (±) and lambda (±) in podocytes (Figures 1e, 1f); IgG1 (++), IgG2 (±), IgG3 (±), and IgG4 (+) in podocytes and mesangium (Figures 1g-1j); M-type phospholipase A2 receptor (PLA2R) negative (IgA, IgM, C1q, and PLA2R immunofluorescence is not shown in Figure 1) [8]. Electron microscopy showed diffuse podocyte foot process effacement, and electron-dense deposits were not evident (Figure 3). Figure 4 shows an immunofluorescence image using structured illumination microscopy (SIM), demonstrating the decreased expression of nephrin and punctuate IgG deposition colocalized with nephrin (Figures 4a-4c). Seventeen months after COVID-19 infection, serum anti-nephrin antibody was negative (163 U/ml (cut-off: 226 U/ml); see Appendices for details of histopathology methods and anti-nephrin antibody measurements), and IgG was 335 mg/dl. Plasmapheresis was performed 12 times. Serum albumin was increased (3.4g/dl), but proteinuria remained severe (43.3 g/gCr). Twenty-one months after the COVID-19 infection, his serum was positive for anti-nephrin antibodies (367 U/ml), serum IgG was 769 mg/dl, and he still required maintenance dialysis therapy.

## Discussion

The present study reports a case of nephrotic syndrome after COVID-19 infection. An initial biopsy showed minimal change disease positive for IgG, and examination of a second biopsy using SIM showed collapsing FSGS with punctuate IgG co-localized with nephrin.

The first serum measurement for anti-nephrin antibodies was negative, probably because of the low serum IgG value (serum IgG = 335 mg/dl), but the patient’s serum was subsequently found to be positive for anti-nephrin antibodies (serum IgG = 769 mg/dl). Watts et al. reported that anti-nephrin antibodies in minimal change disease were present in IgG-positive biopsies and that punctuate IgG features co-localized with nephrin [3]. Shirai et al. showed that anti-nephrin autoantibodies were present in recipient plasma following FSGS recurrence after kidney transplantation [4,9]. In addition, IgG co-localized with nephrin in a one-hour allograft biopsy from a patient with FSGS recurrence after kidney transplantation and in allograft biopsies from patients with FSGS recurrence in a multicenter study [4]. In our case, a second biopsy showed collapsing FSGS by light microscopy, IgG-positive staining in IF, widespread foot process effacement by electron microscopy, but no evidence of electron-dense deposits, indicating anti-nephrin antibody-associated FSGS. To distinguish membranous nephropathy, PLA2R staining was performed, which was negative [8]. SIM showed the colocalization of IgG and nephrin. Furthermore, the serum was positive for anti-nephrin antibodies. Histological results from the first biopsy also showed IgG staining in the podocytes with widespread foot process effacement. These results suggest that collapsing FSGS in the second biopsy and minimal change disease in the first biopsy may have been caused by anti-nephrin antibodies.

There was a discrepancy in IgG staining patterns between Figure 1c and Figure 4a. A polyclonal IgG antibody was used in Figure 1c, and a monoclonal antibody was used in Figure 4 in SIM, which might have been the cause of this discrepancy in IgG immunofluorescence staining features. In Figure 1c, IgG deposition was observed in the mesangium area, as well as in podocytes. A previous report showed that mesangial IgG deposits were present in pediatric minimal change cases, suggesting that IgG deposition in the mesangium predicts a difficult clinical course [10]. However, the detailed pathogenesis of IgG deposition in the mesangium in Figure 1c remains unclear. IgG antibodies other than anti-nephrin antibodies might be involved in the IgG deposition present in the mesangium. In our case, IgG1 and IgG4 were the dominantly stained IgG subclasses. Watts et al. reported that punctuate IgG deposition was IgG1 or IgG4 in minimal change disease [3]. Shirai et al. reported that diverse IgG class patterns were present in cases of recurrent FSGS after kidney transplantation [4]. Further research is needed to assess the relationship between IgG subclass pattern and clinical findings and prognosis in anti-nephrin antibody-associated minimal change disease or FSGS.

Minimal change disease and FSGS are considered to be different expressions of the same progressive lesion [1]. A case of collapsing FSGS following minimal change disease after COVID-19 infection has been reported in the literature [11]. In the present case, the first biopsy indicated minimal change disease, and the second showed a pathological finding of collapsing FSGS. Thus, anti-nephrin antibodies may represent a common etiology for minimal change disease and for collapsing FSGS after COVID-19 infection.

A recent case report used molecular profiling analysis to show that the activation of COVID-19-related cell injury, similar to inflammation and coagulation, was observed in the kidney of a patient with collapsing glomerulopathy associated with COVID-19 [12]. However, our case showed collapsing FSGS with epithelial cell hyperplasia 15 months after COVID-19 infection; therefore, it is unlikely that collapsing FSGS was caused by a short-term COVID-19-induced inflammatory response. COVID-19 infection could have triggered the persistent production of anti-nephrin antibodies, leading to collapsing FSGS lesions. However, further studies are required to elucidate the mechanism by which COVID-19 infection triggers anti-nephrin antibody production.

Our patient was clinically resistant to treatment, and collapsing FSGS with diffuse foot process effacement at the second biopsy suggested a poor treatment response pathologically. A recent report showed that B-cell depleting therapy was effective at controlling disease activity in one case of minimal change disease positive for anti-nephrin antibodies [13]. However, Shirai et al. [4] reported that nine of 11 kidney transplant recipients with FSGS recurrence achieved remission despite treatment with and without plasmapheresis and rituximab. Referring to a previous report [14], Shirai et al. also discussed the unknown cause of different treatment responses and the variety of pathogenic processes of B-cell dysregulation [4]. Further studies are needed to examine the association between COVID-19 infection and B-cell dysregulation.

## Conclusions

In summary, this report showed that the first biopsy indicated minimal change with IgG deposition in the podocytes and mesangium after COVID-19 infection and that the second biopsy showed collapsing FSGS with IgG deposition in the podocytes and mesangium. Immunofluorescence imaging using SIM in the second biopsy showed decreased nephrin expression and punctuate IgG deposition colocalized with nephrin. Thereafter, the serum anti-nephrin antibodies were found to be positive. This case highlights that anti-nephrin antibodies induced collapsing FSGS following minimal change disease, possibly triggered by COVID-19 infection. Further studies are required to elucidate the mechanism by which anti-nephrin antibody production is triggered by COVID-19 infection.

## Appendices

Supplemental Methods

Anti-nephrin Antibody Measurement

Circulating anti-nephrin antibodies were quantified by enzyme-linked immunosorbent assay (ELISA) as previously described [4], except that a commercially available human embryonic kidney-derived recombinant extracellular domain of nephrin was used (17757-H08H; Sino Biological Inc., Wayne, PA, USA). Briefly, Nunc MaxiSorp™ ELISA plates (Thermo Fisher Scientific, Waltham, MA, USA) were coated with 100 ng/well of the recombinant extracellular domain of human nephrin and incubated overnight at 4°C. Uncoated control wells (without antigen) were used to determine nonspecific binding for each sample, allowing for background subtraction. After washing, plates were blocked with 300 µl of Superblock (Thermo Fisher Scientific) for one hour at room temperature and then incubated overnight with patient samples diluted at 1:400. Plates were incubated with goat anti-human IgG Fc (HRP). After washing, 3,3',5,5'-tetramethylbenzidine substrate was added, followed by the stop solution 10 minutes later. Absorbance was measured at 450 nm. Anti-nephrin antibody titers were determined using a standard curve derived from serial two-fold dilutions of the index patient’s sample, which was previously reported [4], with the patient’s titer arbitrarily defined as 1000 units (U)/mL. The cut-off for positivity was defined as the maximum antibody titer in controls from healthy individuals (n = 13), and disease controls from patients with membranous nephropathy (n = 13) and lupus nephritis (n = 4). Serum samples were obtained from patients with membranous nephropathy and lupus nephritis when they presented with proteinuria.

Dual Immunofluorescence Staining Protocol (IgG and Nephrin)

Immunofluorescence studies were performed as previously reported [4]. Briefly, sections 2 µm thick were prepared and incubated with an anti-nephrin antibody (Anti-Human Nephrin (C) Rabbit IgG Affinity Purify, Immuno-Biological Laboratories, Fujioka, Japan) at a dilution of 1:50 and anti-IgG mouse monoclonal antibody (B-MU367UC, BIOGENEX, CA, USA) at a dilution of 1:40. Goat anti-rabbit IgG cross-adsorbed secondary antibody, Alexa Fluor 568, and goat anti-mouse IgG cross-adsorbed secondary antibody, Alexa Fluor 488 (Thermo Fisher Scientific, Waltham, MA) were used at a dilution of 1:1000. Image acquisition was performed using two-dimensional structured illumination microscopy mode with a Nikon microscope (N-SIM; Nikon, Tokyo, Japan), and image reconstruction was carried out using NIS-Elements software (Nikon) based on a previous report [4], with kind support from Dr. Noriko Tokai of the Imaging Core Laboratory (Institute of Medical Science, The University of Tokyo, Tokyo, Japan).
